# Supplementary material for: Glucose Detection of 4-Mercaptophenylboronic Acid-Immobilized Gold-Silver Core-Shell Assembled Silica Nanostructure by Surface Enhanced Raman Scattering
Source: Nanomaterials (Basel). 2021 Apr 8;11(4):948. doi: 10.3390/nano11040948 (PMC8068217; doi:10.3390/nano11040948)
Supplement: Supplementary file 1 [file nanomaterials-11-00948-s001.pdf]

Glucose detection of 4-mercaptophenylboronic acid-gold-silver core-shell assembled silica nanostructure by Surface enhanced Raman scattering.

Xuan-Hung Pham [1,] Bomi Seong [1], Eunil Hahm [1], Kim-Hung Huynh [1], Yoon-Hee Kim [1], Jaehi Kim [1], Sang Hun Lee [2], and Bong Hyun Jun [2,*]

1 Department of Bioscience and Biotechnology, Konkuk University, Seoul 05029, Republic of Korea

2 Department of Chemical and Biological Engineering, Hanbat National University, Daejeon 34158, Republic of Korea.

* Correspondence: bjun@konkuk.ac.kr (B-H. J); Tel.: +82-2-450-0521 (B.H.J.)

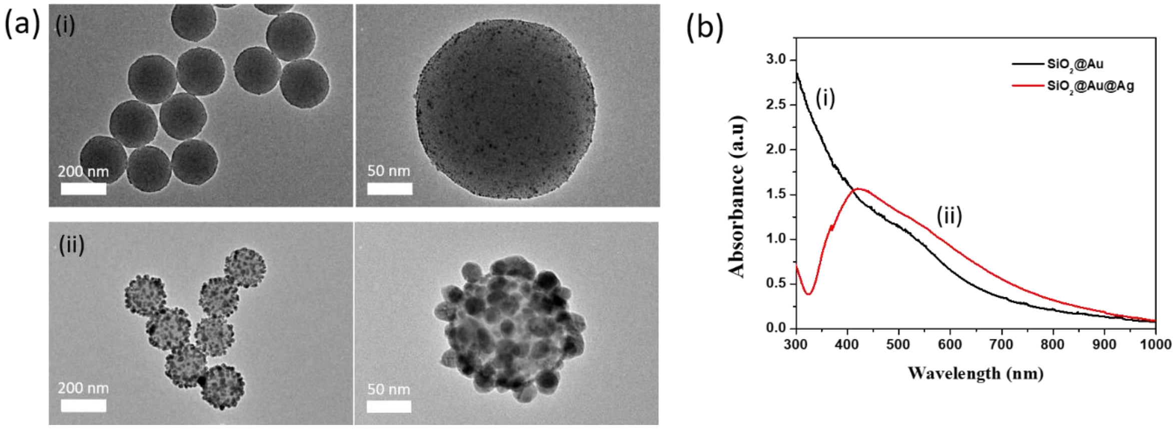

Figure S1. (a) TEM images and (b) UV-Vis spectra of (i) SiO_2@Au (1 mg/mL) and (ii) SiO_2@Au@Ag nanostructures (20 μg/mL) synthesized at 2 mg SiO_2@NH_2 and 300 mM Ag^+ concentration.

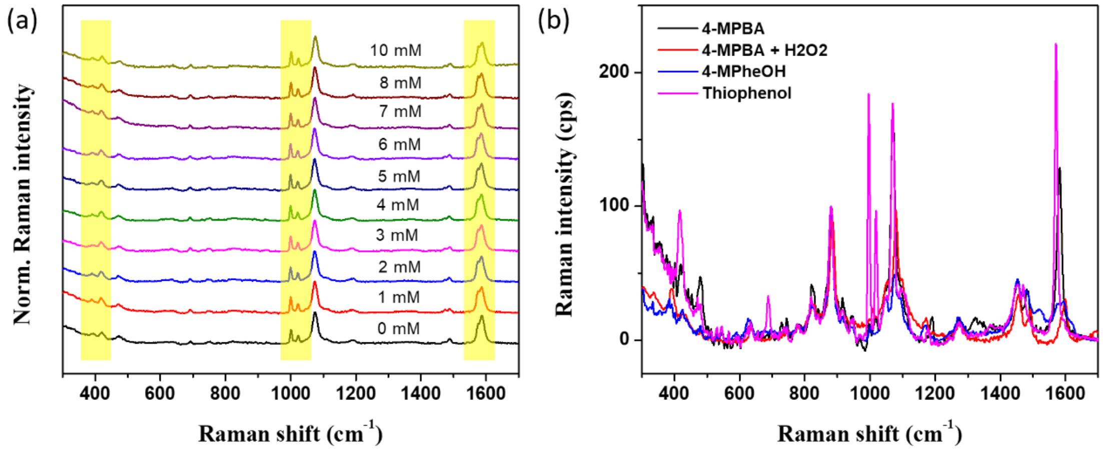

Figure S2. (a) SERS spectra of SiO_2@Au@Ag@4-MPBA in PBST containing difference concentration of glucose (0-10 mM). (b) SERS spectra of SiO_2@Au@Au in ethanol in the presence of 50 μM 4-mercaptophenylboronic acid, 50 μM 4-mercaptophenylboronic acid + 1000 μg/mL H_2O_2, 1 mM 4-mercaptophenol, 1 mM thiophenol.

Table S1. Raman frequencies and assignments of 4-MPBA and 4-MPheOH in EtOH and PBST

Assignment	4-MPBA in EtOH	4-MPheOH in EtOH	4-MPBA in PBST
8a(a_1), ν_{CC}		1597	
	1583	1583	1583
			1578
19a(a_1), ν_{CC}	1484	1484	1484
ν_{BO}	1346		
3(b_2), $\beta_{CH} + \beta_{BOH}$	1286		
9a(a_1), $\beta_{CH} + \beta_{BOH}$	1192		1192
		1170	
1(a_1), $\beta_{CCC} + \nu_{CS}$	1077		1077
18a(a_1), β_{CH}			1025
12(a_1), β_{CCC}			999
10a(a_2), γ_{CH}	820	824	824
11(b_1), γ_{CH}	744	744	752
	728		
6a(a_1), $\beta_{CCC} + \nu_{CS}$	690		693
ν_{CS}	632	625	638
6b(b_1), β_{CCC}			612
16b(b_1), β_{CCC}	473	473	473
7a(a_1), $\beta_{CCC} + \nu_{CS}$	417	417	417
		390	

ν: stretching; β: in-plane bending; γ: out-plane bending.

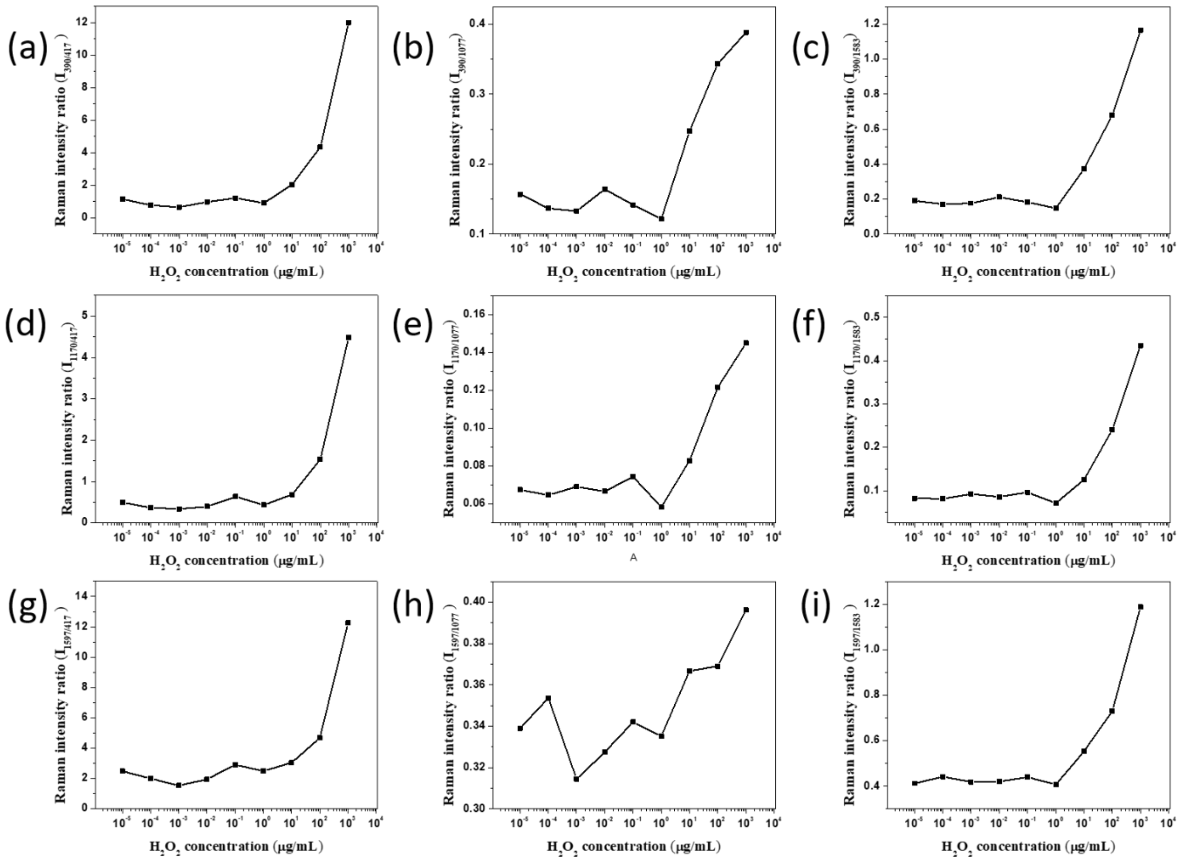

Figure S3. Normalized Raman intensity ratio of 20 μg SiO_2@Au@Ag@4-MPBA in PBST containing various concentration of H_2O_2 in the range of 10^{-6} – 10^{3} μg/mL at (a) 390/417; (b) 390/1077; (c) 390/1583; (d) 1170/417; (e) 1170/1077; (f) 1170/1583; (g) 1597/417; (h) 1597/1077 and (f) 1597/1583.

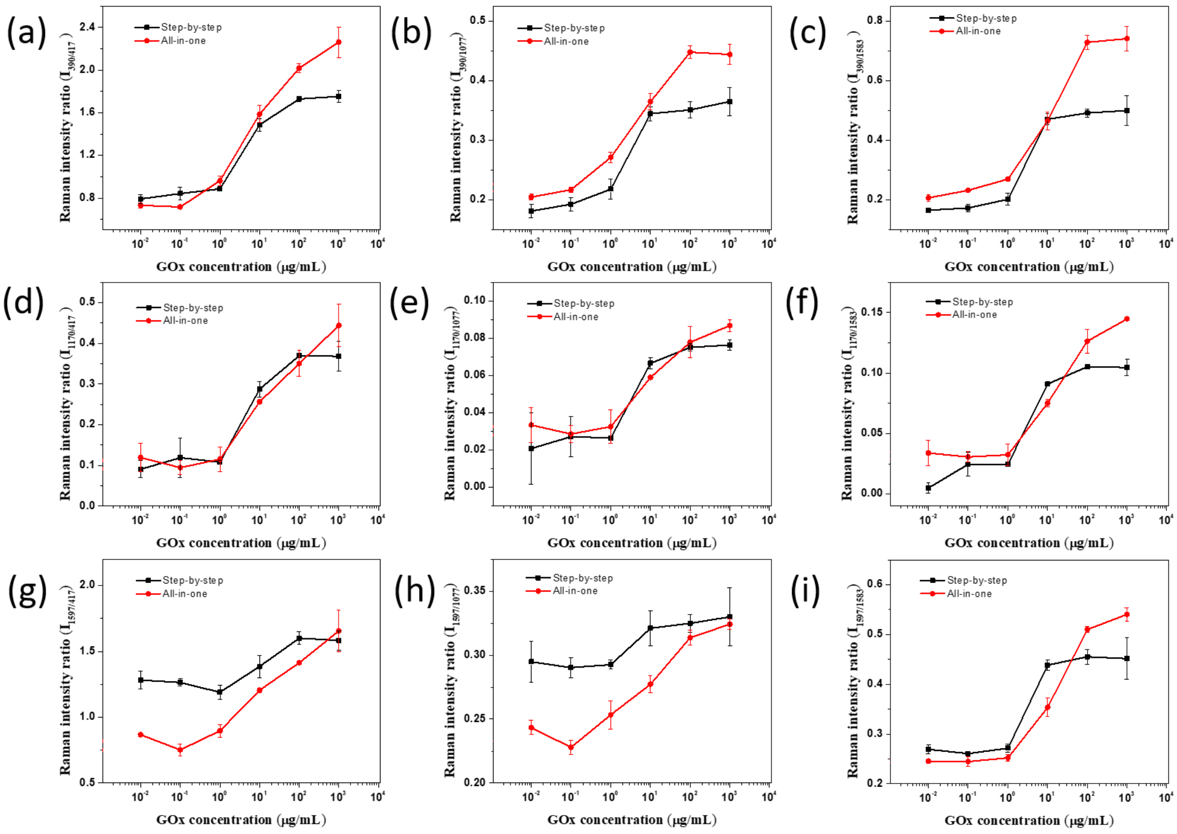

Figure S4. Normalized Raman intensity ratio of 20 μg SiO_2@Au@Ag@4-MPBA in PBST containing 5 mM glucose and difference concentration of glucose oxidase in the range of 10^{-2} – 10^{3} μg/mL at (a) 390/417; (b) 390/1077; (c) 390/1583; (d) 1170/417; (e) 1170/1077, (f) 1170/1583, (g) 1597/417, (h) 1597/1077 and (i) 1597/1583.

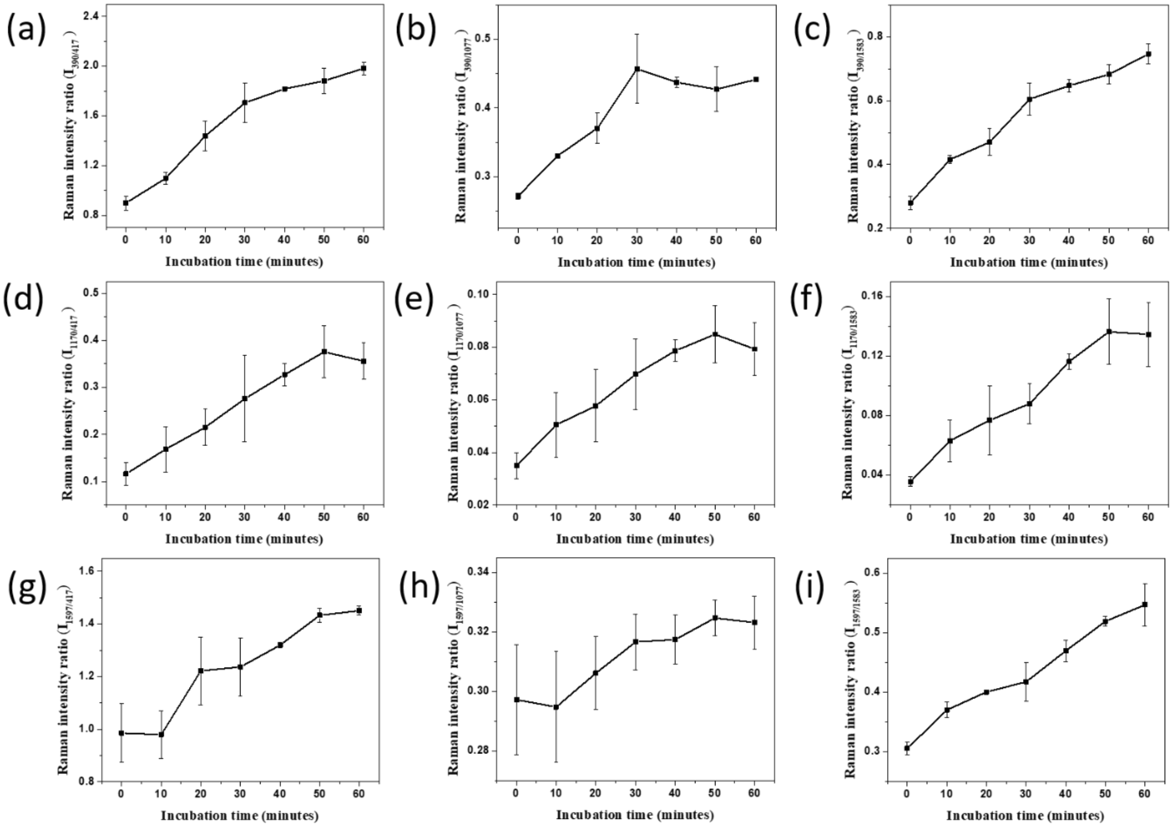

Figure S5. Normalized Raman intensity ratio of 20 μg SiO_2@Au@Ag@4-MPBA in PBST containing 5 mM glucose and 100 μg/mL glucose oxidase at different incubation time at (a) 390/417; (b) 390/1077; (c) 390/1583; (d) 1170/417; (e) 1170/1077, (f) 1170/1583, (g) 1597/417, (h) 1597/1077 and (i) 1597/1583.

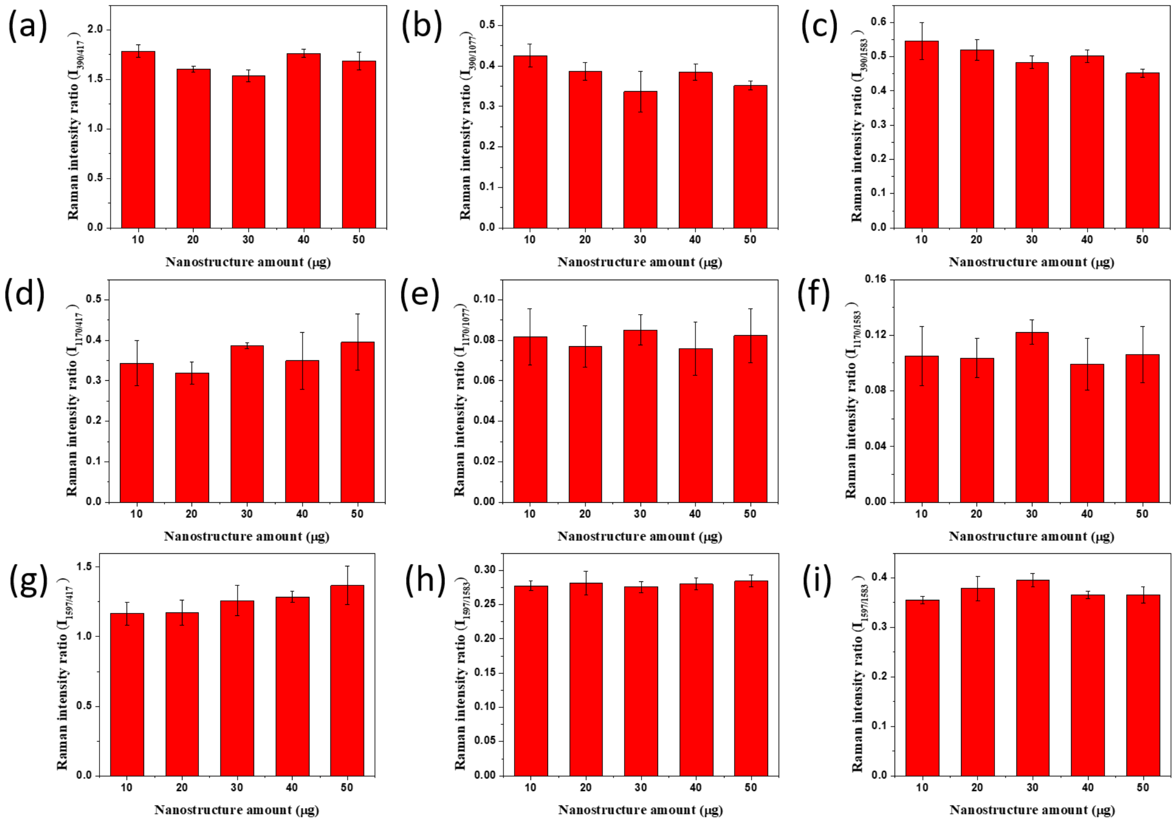

Figure S6. Normalized Raman intensity ratio of SiO_2@Au@Ag@4-MPBA using different amount of SiO_2@Au@Ag (10 - 50 μg) in PBST containing 5 mM glucose, 100 μg/mL glucose oxidase for 1 hr at (a) 390/417; (b) 390/1077; (c) 390/1583; (d) 1170/417; (e) 1170/1077, (f) 1170/1583, (g) 1597/417, (h) 1597/1077 and (i) 1597/1583.

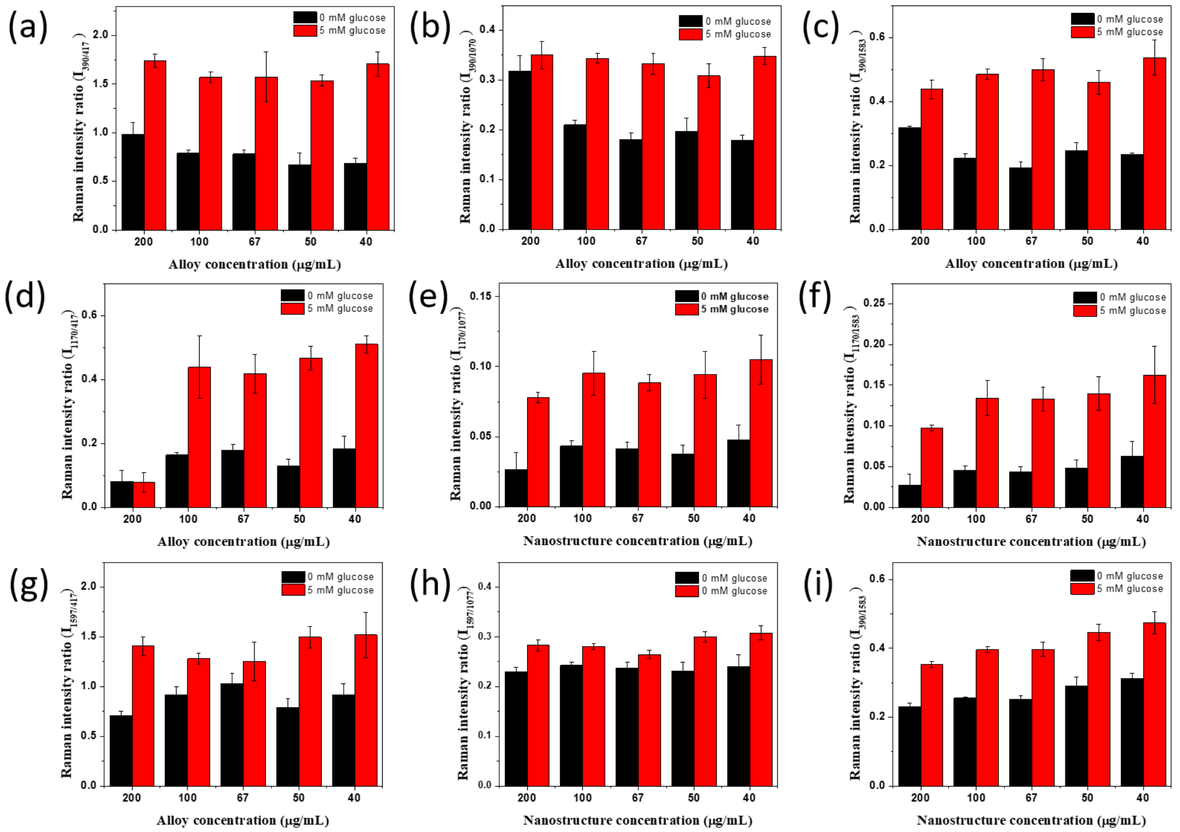

Figure S7. Normalized Raman intensity ratio of 20 µg SiO_2@Au@Ag@4-MPBA in PBST containing 5 mM glucose, 100 µg/mL glucose oxidase for 1 h and measure at different concentration of SiO_2@Au@Ag at (a) 390/417; (b) 390/1077; (c) 390/1583; (d) 1170/417; (e) 1170/1077, (f) 1170/1583, (g) 1597/417, (h) 1597/1077 and (i) 1597/1583.

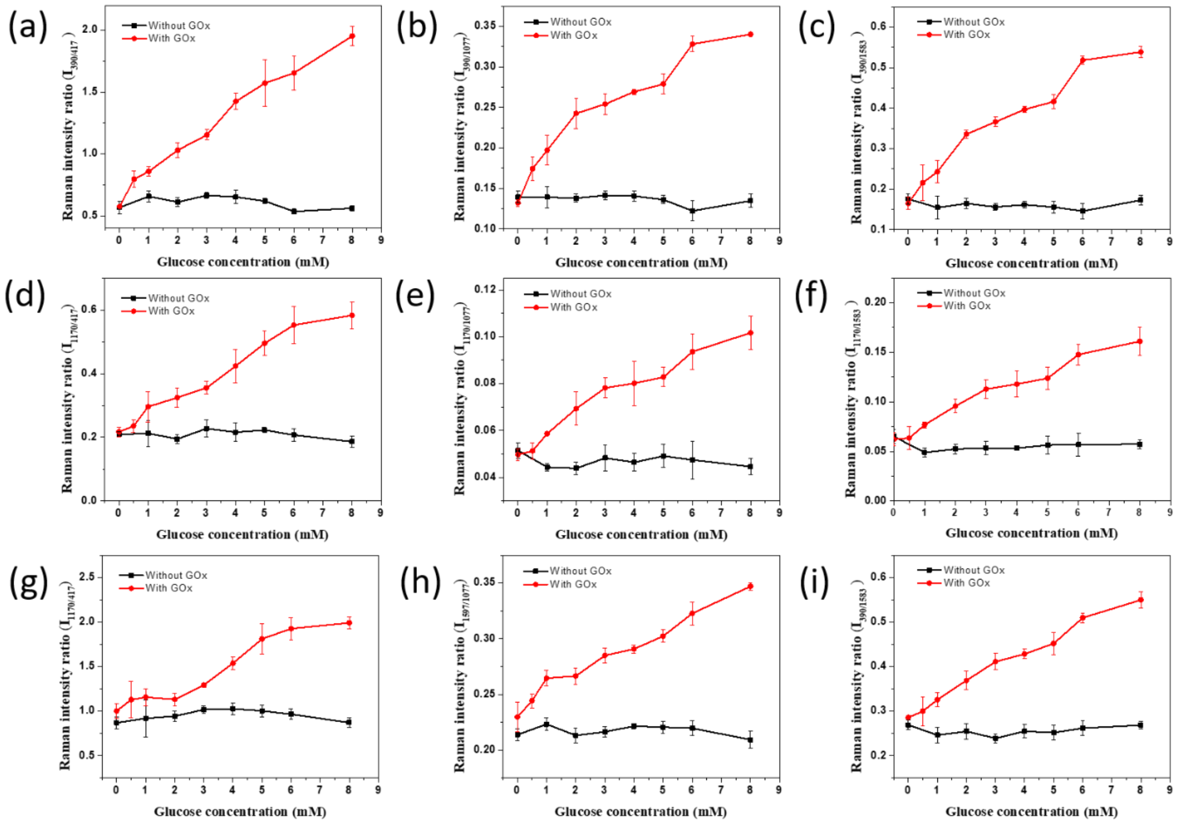

Figure S8. Glucose detection by SiO_2@Au@Au@4-MPBA at optimized condition of 20 µg SiO_2@Au@Ag, 100 µg/mL glucose oxidase concentration for 1h and Raman measurement at 67 µg/mL SiO_2@Au@Ag in PBST at (a) 390/417; (b) 390/1077; (c) 390/1583; (d) 1170/417; (e) 1170/1077, (f) 1170/1583, (g) 1597/417, (h) 1597/1077 and (i) 1597/1583.

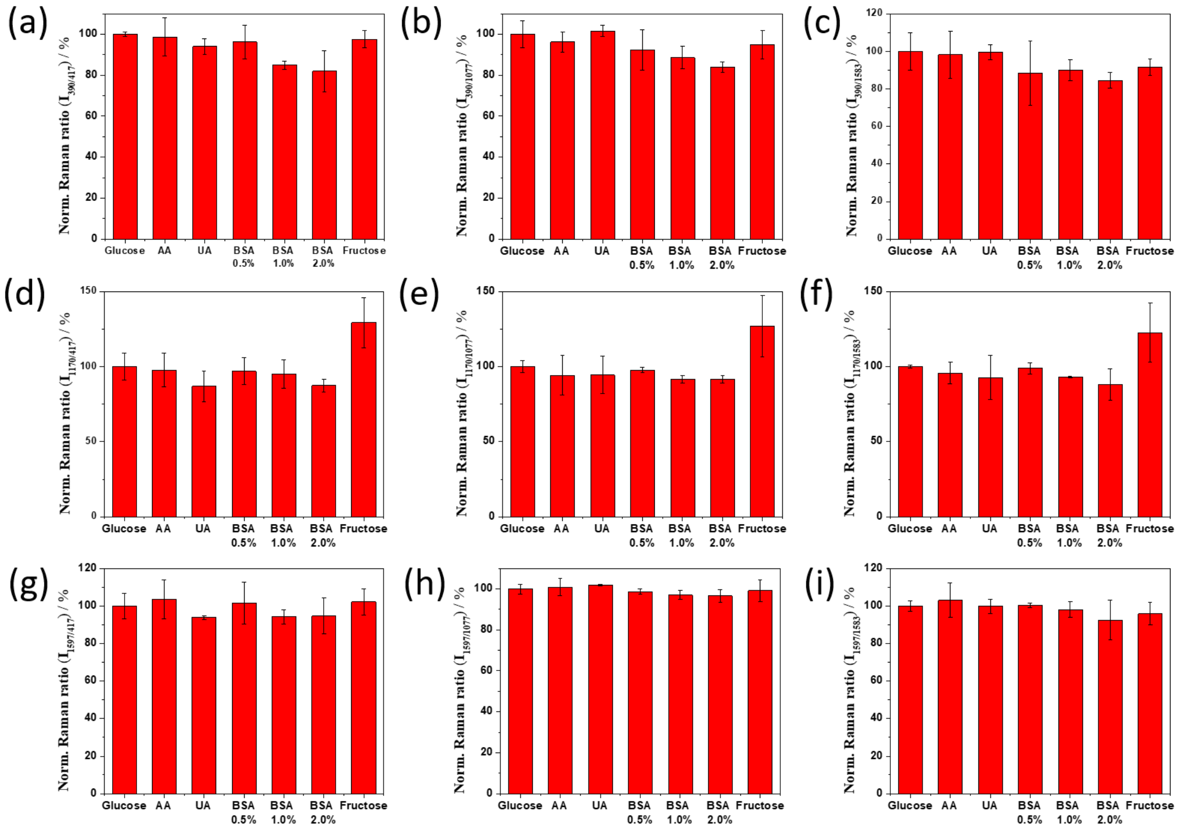

Figure S9. Effect of interferences on SERS signal of SiO_2@Au@Ag@4-MPBA in 5 mM **g**lucose by SiO_2@Au@Au@4-MPBA at optimized condition of 20 µg SiO_2@Au@Ag, 100 µg/mL glucose oxidase concentration for 1h and Raman measurement at 67 µg/mL SiO_2@Au@Ag in PBST at (a) 390/417; (b) 390/1077; (c) 390/1583; (d) 1170/417; (e) 1170/1077, (f) 1170/1583, (g) 1597/417, (h) 1597/1077 and (i) 1597/1583.

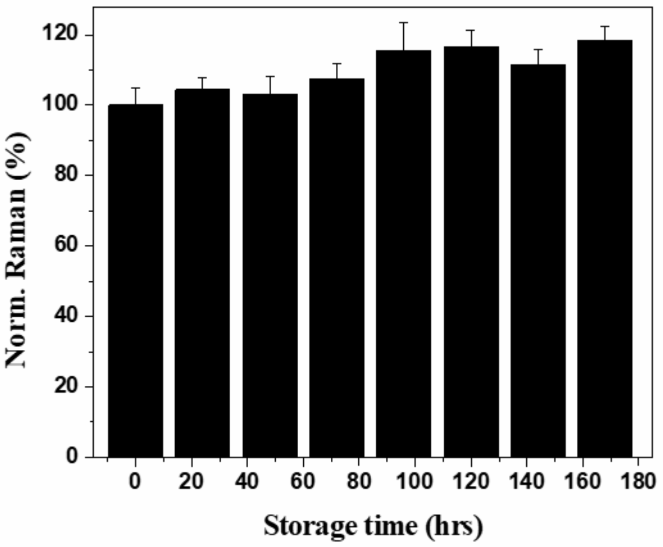

Figure S10. Long-term storage of 200 μg/mL SiO_2@Au@Ag@4-MPBA at 4^oC in ethanol solution.
